# Asymmetric Total Synthesis of Ieodomycin B

**DOI:** 10.3390/md15010017

**Published:** 2017-01-18

**Authors:** Shuangjie Lin, Jianting Zhang, Zhibin Zhang, Tianxiang Xu, Shuangping Huang, Xiaoji Wang

**Affiliations:** 1 School of Pharmacy, Jiangxi Science and Technology Normal University, Fenglin Road 605, Nanchang 330013, China; linsj0317@163.com (S.L.); 13979134167@163.com (J.Z.); haozhangzhibin@163.com (Z.Z.); 18279111449@163.com (T.X.); 2 School of Life Science, Jiangxi Science and Technology Normal University, Fenglin Road 605, Nanchang 330013, China

**Keywords:** antimicrobial, total synthesis, ieodomycin B, chelation-controlled Mukaiyama aldol reaction

## Abstract

Ieodomycin B, which shows in vitro antimicrobial activity, was isolated from a marine *Bacillus* species. A novel asymmetric total synthetic approach to ieodomycin B using commercially available geraniol was achieved. The approach involves the generation of 1,3-*trans*-dihydroxyl at C-3 and C-5 positions via a Crimmins-modified Evans aldol reaction and a chelation-controlled Mukaiyama aldol reaction of a *p*-methoxybenzyl-protected aldehyde, as well as the generation of a lactone ring in a deprotection–lactonization one-pot reaction.

## 1. Introduction

**Figure 1:**
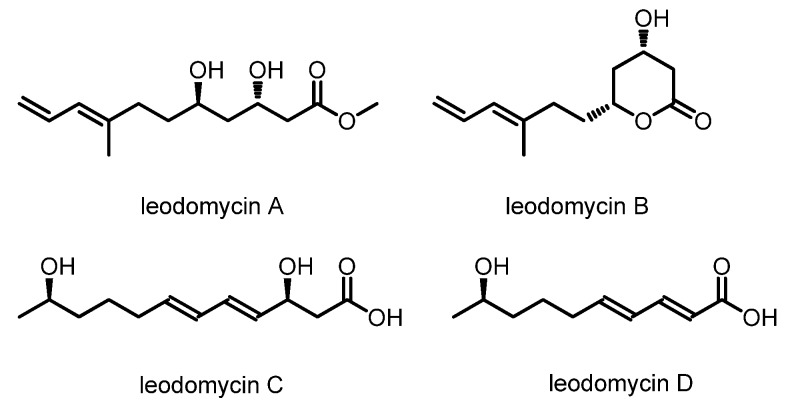
Chemical structures of ieodomycins A–D.

Natural products are an important resource for drug discovery. Isolation, identification, and syntheses of these products or their derivatives, as well as their subsequent biological studies are of interest. In recent years, marine natural products have attracted much interest from scientists. A vast range of chemically diverse biologically active compounds that have antibacterial and anticancer activities have been discovered [1,2,3,4]. The increasingly serious problem of bacterial resistance toward antibiotics has made the search for new antimicrobial agents from natural sources urgent. Consequently, novel and effective antimicrobial compounds have attracted much interest of scientists since its isolation. In 2011, the antimicrobial compounds ieodomycins A–D were first isolated by Shin and coworkers from the EtOAc extract of a marine strain of *Bacillus* species. Through NMR and HRMS analysis and modified Mosher’s method, the planar structures and the absolute configurations were determined, as shown in Figure 1. Subsequent in vitro antimicrobial experiments showed that all of these compounds possess a broad spectrum of antimicrobial activities. They are active against *Bacillus subtilis* and *Escherichia coli*, with minimum inhibitory concentrations (MICs) of 32–64 μg/mL [5].

**Scheme 1:**
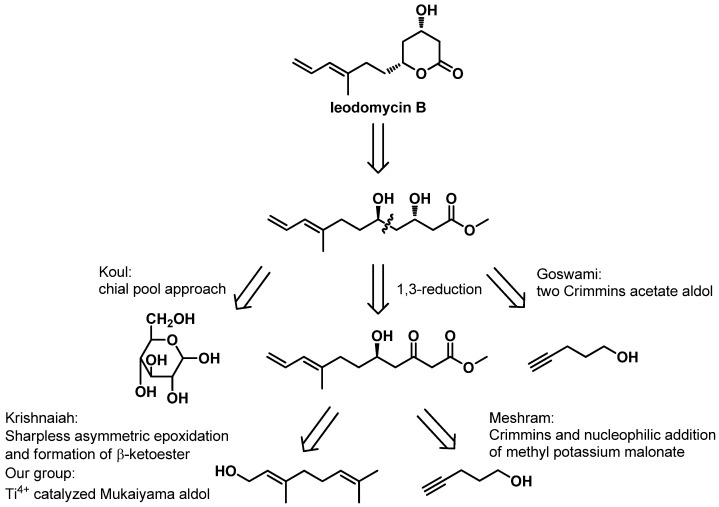
Reported total syntheses of ieodomycin B.

Ieodomycin B has a potential therapeutic application as an antimicrobial agent, and it has an extremely low isolation yield (3.4 mg of ieodomycin B was obtained from 100 L of culture broth) and the greatest complexity among all ieodomycins. Consequently, it has attracted attention from chemical researchers. Several approaches to the synthesis of antimicrobial fatty acids have been developed. As depicted in Scheme 1, four groups besides our own have achieved different total syntheses of ieodomycin B in the same year. Koul first reported the total synthesis of ieodomycin B using the chiral-pool approach starting from d-glucose, which consists of more than 15 steps [6]. Almost simultaneously, Krishnaiah published another stereoselective total synthetic route for ieodomycin B starting from geraniol, which also requires 15 steps [7]. In Krishnaiah’s route, a protocol that includes Sharpless asymmetric epoxidation-epoxide opening and subsequent 1,3-reduction is used to construct the chiral hydroxyl groups at the C-3 and C-5 positions. Meshram described a novel protection-free, stereoselective, eight-step synthesis of ieodomycin B from commercially available 4-pentyne-1-ol [8]. This route involves a Crimmins-modified Evans aldol reaction and a nucleophilic addition of the potassium salt of monomethyl malonate, forming the δ-hydroxyl β-keto ester. It then ends in a 1,3-reduction, affording the desired chiral centers. Goswami and coworkers achieved ieodomycin B in nine linear steps starting from the same alcohol. In their route, they used the Crimmins-modified Evans aldol reaction twice to construct the two chiral hydroxyls [9]. Recently, our group reported a short total synthesis of ieodomycin B involving seven steps [10]. In this approach, we used Ti^4+^-catalyzed asymmetric Mukaiyama aldol reaction and the subsequent 1,3-induced reduction to construct the chiral centers. However, its enantioselectivity is only 88% ee. This unsatisfactory result prompted us to develop an alternative route to ieodomycin B.

In continuation of our studies on developing a new approach toward the synthesis of lactones and on obtaining the natural product at a higher optical purity, we focused on the total synthesis of ieodomycin B. Although the chemical structure of ieodomycin B appears to be simple, a novel and effective protocol to its total synthesis is important. It is well-known that chelation-controlled Mukaiyama aldol addition using alkoxy groups such as *p*-methoxybenzyl (PMB) ether or benzyl (Bn) ether is a very important methodology for the highly diastereoselective construction of the *syn*/*anti*-diol unit (for an example, see [11]). Using this methodology, we developed in the present study a total synthesis of ieodomycin B using a Crimmins-modified Evans aldol reaction. This reaction is followed by a Mukaiyama aldol reaction induced by PMB ether to selectively construct C-5 and C-3 chiral centers.

## 2. Results

**Scheme 2:**
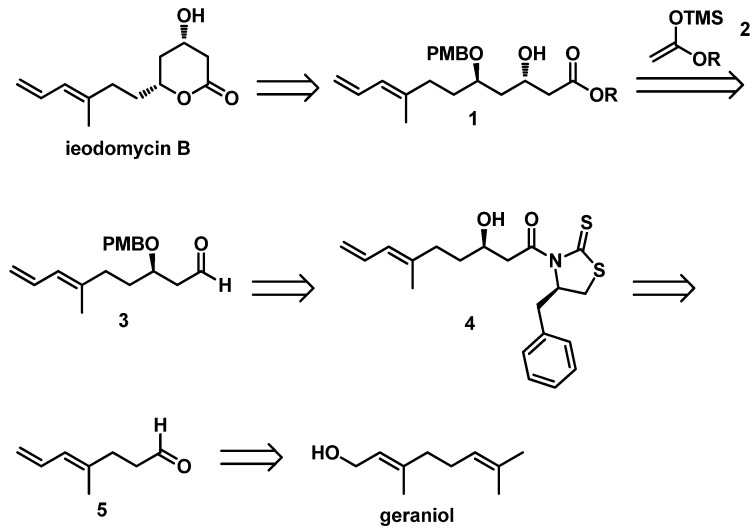
Retrosynthetic analysis of ieodomycin B.

On the basis of the above considerations, we utilized the 1,3-asymmetrically inductive Mukaiyama aldol reaction in the total synthesis of ieodomycin B. Therefore, we performed our retrosynthetic analysis based on this strategy, as depicted in Scheme 2. We envisioned retrosynthetically that the target molecule ieodomycin B could be prepared from the intermediate **1**, which is suitable for disconnection from the protected aldehyde **3** and silyl enol ether **2** via the 1,3-inductive Mukaiyama aldol reaction. We noticed that **1** has *trans* configuration. Considering that the chelation-controlled step and deprotection are simple, we decided to protect the hydroxyl in **1** using PMB, in accordance with the studies of Munro et al. [12,13]. The total synthesis was mainly focused on the formation of the precursor **3**. We propose that **3** can be obtained through a few conversion steps from **4**, which can be prepared by a Crimmins-modified Evans aldol reaction of aldehyde **5**. Compound **5** could be easily prepared from geraniol.

**Scheme 3:**
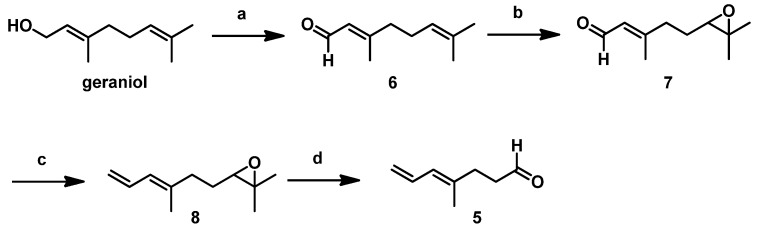
Synthetic route of aldehyde **5**. Reagents and conditions: (**a**) (COCl)_2_, DMSO, Et_3_N, dichloromethane (DCM), −78 °C to 0 °C, 98%; (**b**) *m*-CPBA, DCM, 0 °C, 85%; (**c**) Ph_3_PCH_3_Br, *n*-BuLi, tetrahydrofuran (THF), 0 °C, 75%; (**d**) HIO_4_, THF/H_2_O (5:3), 0 °C, 84%.

The detailed synthetic route for **5** starting from commercially available geraniol followed procedures described in our previous report [10]. As shown in Scheme 3, geraniol underwent Swern oxidation, regioselective epoxidation of the isolated double bond using *m*-CPBA, and subsequent Wittig olefination with Ph_3_P=CH_2_ to the stable epoxide **8**. Using HIO_4_, we cleaved the resulting epoxide to afford the low-boiling-point **5**, which we then immediately subjected to the Crimmins-modified Evans aldol reaction after aborative manipulation.

**Scheme 4:**
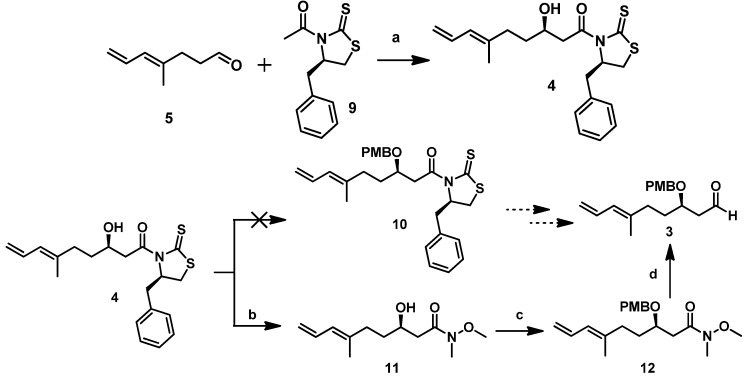
Synthetic route of the intermediate **3**. Reagents and conditions: (**a**) TiCl_4_, DIPEA, DCM, −40 °C to −78 °C, 55%; (**b**) MeONHMe•HCl, imidazole, DCM, rt, 68%; (**c**) NaH, PMBBr, DMF, −15 °C, 67%; (**d**) DIBAL-H, DCM, −78 °C, 61%.

With **5** in hand, secondary alcohol **4** could be obtained by using a Crimmins-modified Evans aldol addition with the acetylthiazolidine thione **9**. In the presence of TiCl_4_ and (i-Pr)_2_NEt (DIPEA), titanium enolate generated from **9** reacted with **5**, forming the predominant desired isomer **4** in excellent yield [8]. As described above, we initially attempted to protect the hydroxyl in **4** using the PMB group under certain conditions, but we could not obtain the desired compound **10**. Therefore, we proceeded to convert **4** to a Weinreb amide and then protected it. Direct amidation of **4** with MeONHMe•HCl catalyzed by imidazole yielded Weinreb amide **11**. Subsequent treatment of the latter with PMBBr in the presence of NaH afforded the PMB ether **12** in a 67% yield (Scheme 4). Subsequently, **12** was reduced with DIBAL-H at −78 °C for 1 h to produce the Mukaiyama aldol precursor aldehyde fragment **3** in about a 61% yield.

**Scheme 5:**
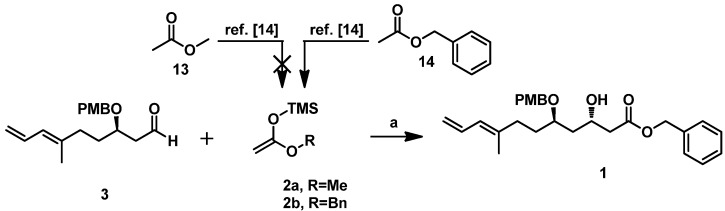
Synthetic route of the intermediate **1**. Reagents and conditions: (**a**) Ti(O^i^Pr)_2_Cl_2_, PhCH_3_, −78 °C, 72%.

With aldehyde **3** in hand, our next objective was to construct the 1,3-*trans* diol unit. We retrosynthetically devised a Mukaiyama aldol reaction induced by a chiral PMB ether in C-5 to selectively construct the C-3 chiral hydroxyl. In our previous study, we found that the mixed titanium species Ti(O^i^Pr)_2_Cl_2_ can effectively induce a chelation-controlled Mukaiyama aldol reaction between the β-alkoxy aldehyde and silyl enol ether, affording the 1,3-*trans* diol in high yield and with high diastereoselectivity. Meanwhile, common Lewis acids such as BF_3_•OEt_2_, SnCl_4_, TiCl_4_, and MgBr_2_•OEt_2_ afforded 1,3-*trans* diol in a low to moderate yield. Thus, we used Ti(O^i^Pr)_2_Cl_2_ in the key Mukaiyama aldol condensation. Considering the possibility of obtaining ieodomycin A and the simplicity of the lactonization, we first attempted to use the ((1-methoxyvinyl)oxy)trimethylsilane **2a** as the nucleophilic reagent for reaction with **3**, but failed. We could hardly obtain the related silyl enol ether of methyl acetate upon treatment of methyl acetate with lithium diisopropylamide (LDA) and trimethyl chlorosilane (TMSCl). Believing that the ester group effects the reaction, we switched the methyl to the benzyl group and thus obtained the desired silyl enol ether **2b** [14]. Reaction of the unpurified crude product of silyl–enol etherification of **2b** with **3** was carried out at −78 °C in the presence of the catalyst Ti(O^i^Pr)_2_Cl_2_ (freshly prepared with TiCl_4_ and Ti(O^i^Pr)_4_ at 1:1 ratio in the solvent DCM at 0 °C). This step readily produced the desired 1,3-*anti* product **1** as the single isomer in about a 72% yield (Scheme 5).

**Figure 2:**
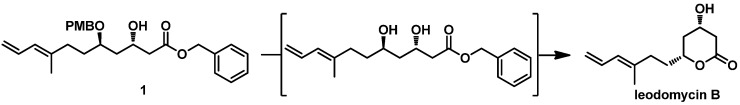
Deprotection and lactonization to ieodomycin B.

Our final procedure was the removal of the PMB protective group. We originally conducted a stepwise protocol consisting of deprotection and lactonization to obtain ieodomycin B. However, when we used typical reagents such as *p*-TsOH, PPTS, DDQ, and CAN, we observed only fuzzy spots in thin-layer chromatography (TLC) along with a decomposition of **1**. Finally, we found that brief treatment with trifluoroacetic acid (TFA) in DCM at 0 °C afforded ieodomycin B in a 50% yield (Figure 2 and Table 1). Extending the treatment, however, resulted in decomposition and low yield.

**Figure 3:**
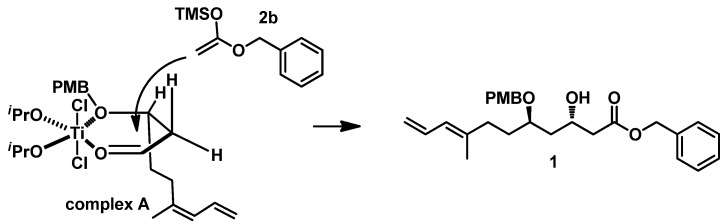
Mechanism of the Mukaiyama aldol addition induced by *p*-methoxybenzyl (PMB) ether.

On the basis of Munro’s work [12], we propose that the highly diastereoselective formation of 1,3-*trans* diol arises from the following mechanism (described in Figure 3): Titanium first forms a chelated complex A with the aldehyde carbonyl in **3** and the ether oxygen of hydroxyl protected by PMB, and the diene group in its preferred conformation occupies a pseudoaxial position. Subsequently, the silyl enol ether ((1-(benzyloxy)vinyl)oxy)trimethylsilane **2b** attacks from the less-hindered side, which is opposite to the diene group, giving the *anti*-diol product **1**.

## 3. Experimental Section

### 3.1. General

All reactions were carried out under N_2_ atmosphere with dry solvents unless otherwise noted and monitored via thin-layer chromatography (TLC) carried out on 0.25 mm silica gel plates (60F-254). Silica gel (200–300 mesh) for flash column chromatography was supplied by Qingdao Marine chemical factory in Qingdao, China. Anhydrous toluene and tetrahydrofuran (THF) was distilled from sodium-benzophenone. Dichloromethane (DCM) and *N*,*N*-dimethyl formamide (DMF) was distilled from CaH_2_. Yield refers to chromatographic and spectroscopic ^1^H and ^13^C NMR, unless otherwise stated. NMR spectra were recorded on a Bruker AV 400 NMR spectrometer (Bruker, Fällanden, Switzerland) (^1^H: 400 MHz, ^13^C: 100 MHz). High-resolution mass spectra were obtained from an Applied Biosystems Q-Star Elite MALDI-TOF mass spectrometer (Applied Biosystems, Carlsbad, CA, USA). Optical rotations were measured on a Rudolph Autopol IV automatic polarimeter (Rudolph, Hackettstown, NJ, USA) in CHCl_3_ at 25 °C.

### 3.2. (R,E)-1-((R)-4-Benzyl-2-thioxothiazolidin-3-yl)-3-hydroxy-6-methylnona-6,8-dien-1-one *(**4**)*

To a solution of thiazolidinethione **9** (4.8 g, 19.3 mmol, 1.2 equiv) in anhydrous DCM (60 mL) TiCl_4_ (3.2 mL, 29.0 mmol, 1.8 equiv) was added dropwise at −40 °C, and the resultant yellowish slurry was stirred for 5 min at the same temperature. Then, DIPEA (4.1 mL, 29.0 mmol, 1.8 equiv) was added slowly. The deep reddish solution was stirred for another 1 h at −40 °C before being cooled to −78 °C, and aldehyde **5** (2.0 g, 16.1 mmol, 1.0 equiv) in DCM (24 mL) was added via a cannula. Stirring of the mixture continued at −78 °C for 20 min and was quenched by saturated aqueous NH4Cl (20 mL). It was then extracted with DCM (3 × 80 mL), washed with brine, dried over anhydrous Na_2_SO_4_, filtered, and concentrated in vacuo. Purification via silica gel column chromatography (petroleum ether/EtOAc = 20:1) yielded compound **4** (3.3 g, 55%) as a light yellow oil.

^1^H NMR (400 MHz, CDCl_3_), δ 7.28–7.27 (m, 2H), 7.23–7.19 (m, 3H), 6.51 (dt, *J* = 16.8 Hz, 12.0 Hz, 1H), 5.84 (d, *J* = 10.4 Hz, 1H), 5.38–5.32 (m, 1H), 5.05 (d, *J* = 16.8, 1H), 4.93 (d, *J* = 10.4 Hz, 1H), 4.11–4.06 (m, 1H), 3.60 (dd, *J* = 17.6 Hz, 2.4 Hz, 1H), 3.28 (dd, *J* = 11.5 Hz, 7.4 Hz, 1H), 3.12 (dd, *J* = 13 Hz, 3.1 Hz, 1H), 3.05 (dd, *J* = 17.7 Hz, 9.4 Hz, 1H), 2.97 (dd, *J* = 13 Hz, 10.4 Hz, 1H), 2.84 (d, *J* = 11.6 Hz, 1H), 2.71 (s, 1H), 2.21–2.12 (m, 1H), 2.12–2.08 (m, 1H), 1.72 (s, 3H), 1.67–1.53 (m, 2H); ^13^C NMR (CDCl_3_, 100 MHz) δ 201.4, 173.1, 138.6, 136.3, 133.2, 129.4, 128.9, 127.2, 125.9, 115.0, 68.2, 67.5, 45.8, 36.8, 35.6, 34.3, 32.0, 16.6.

### 3.3. (R,E)-3-Hydroxy-N-methoxy-N,6-dimethylnona-6,8-dienamide *(**11**)*

To a solution of compound **4** (3.0 g, 8.0 mmol, 1.0 equiv) in DCM (45 mL), MeONHMe•HCl (3.12 g, 32.0 mmol, 4.0 equiv) and imidazole (2.72 g, 40.0 mmol, 5.0 equiv) were added, and the resultant mixture was stirred at room temperature overnight. When it was clear via TLC that compound **4** had been consumed, the reaction was quenched with a saturated aqueous NH_4_Cl solution (20 mL), and the resultant mixture was extracted with DCM (3 × 80 mL). The organic layer was then washed with brine, dried over anhydrous Na_2_SO_4_, filtered, and concentrated under reduced pressure. Purification of the residue by silica gel column chromatography (petroleum ether/EtOAc = 3:1) yielded the desired Weinreb amide **11** (1.23 g, 68%) as a yellow oil.

[α${]}_{D}^{25}$ = +42.4 (*c* = 1.5, CHCl_3_); ^1^H NMR (400 MHz, CDCl_3_) δ 6.56 (dt, *J* = 16.8 Hz, 10.7 Hz, 1H), 5.89 (d, *J* = 10.8 Hz, 1H), 5.09 (d, *J* = 16.8, 1H), 4.98 (d, *J* = 10.0 Hz, 1H), 4.04–3.96 (m, 1H), 3.83–3.80 (m, 1H), 3.68 (s, 3H), 3.19 (s, 3H), 2.69–2.65 (m, 1H), 2.50–2.43 (m, 1H), 2.34–2.20 (m, 1H), 2.20–2.10 (m, 1H), 1.77 (s, 3H), 1.74–1.52 (m, 2H); ^13^C NMR (CDCl_3_, 100 MHz) δ 173.8, 139.0, 133.2, 125.7, 114.8, 67.5, 61.2, 38.1, 35.6, 34.6, 31.8, 16.6. HRMS (ESI): *m*/*z* calcd. for C_12_H_21_NO_3_Na [M + Na]^+^ 250.1414, found 250.1416.

### 3.4. (R,E)-N-Methoxy-3-((4-methoxybenzyl)oxy)-N,6-dimethylnona-6,8-dienamide *(**12**)*

NaH (0.21 g, 5.28 mmol, 1.5 equiv, 60% in mineral oil) was added to the solution of compound **11** (0.80 g, 3.52 mmol, 1.0 equiv) in DMF (12 mL) at −15 °C and PMB-Br (1.03 mL, 7.04 mmol, 2.0 equiv, stabilized with 5 wt % K_2_CO_3_). The suspension was stirred at −15 °C for 1.5 h until it was observed via TLC that compound **11** was consumed; then, it was poured onto H_2_O (5 mL) and ether (3 × 40 mL). The layers were separated and the organic layer was then washed with brine, dried over anhydrous Na_2_SO_4_, filtered, and concentrated at reduced pressure. The residue was purified via silica gel column chromatography (petroleum ether/EtOAc = 10:1) to yield PMB ether **12** (0.82 g, 67%) as a yellow oil.

[α${]}_{D}^{25}$ = −7.5 (*c* = 1, CHCl_3_); ^1^H NMR (400 MHz, CDCl_3_) δ 7.26 (d, *J* = 8.0 Hz, 2H), 6.86 (d, *J* = 8.0 Hz, 2H), 6.48 (dt, *J* = 16.8 Hz, 10.5 Hz, 1H), 5.85 (d, *J* = 10.8 Hz, 1H), 5.08 (d, *J* = 16.8, 1H), 4.98 (d, *J* = 10.1 Hz, 1H), 4.47 (q, *J* = 10.8 Hz, 2H), 3.98–3.92 (m, 1H), 3.79 (s, 3H), 3.66 (s, 3H), 3.19 (s, 3H), 2.86 (dd, *J* = 14.6, 6.2 Hz, 1H), 2.48 (dd, *J* = 15.0, 5.3 Hz, 1H), 2.25–2.07 (m, 2H), 1.75 (s, 3H), 1.80–1.66 (m, 2H); ^13^C NMR (CDCl_3_, 100 MHz) δ 159.1, 139.1, 133.3, 130.8, 129.4, 125.6, 114.7, 113.7, 75.6, 71.6, 61.3, 55.2, 37.2, 35.4, 33.1, 29.7, 16.6. HRMS (ESI): *m*/*z* calcd. for C_20_H_29_NO_4_Na [M + Na]^+^ 370.1989, found 370.1989.

### 3.5. (R,E)-3-((4-Methoxybenzyl)oxy)-6-methylnona-6,8-dienal *(**3**)*

DIBAL-H (1.20 mL, 1.80 mmol, 1.5 M in toluene, 1.3 equiv) was added dropwise to a solution of the Weinreb amide **12** (0.57 g, 1.64 mmol, 1.0 equiv) in freshly distilled DCM (25 mL) at −78 °C. Stirring of the reaction continued at −78 °C for 1 h until it was observed via TLC that compound **12** had been consumed. Then, it was quenched with a saturated aqueous NaCl solution (5 mL). The mixture was extracted with DCM (3 × 20 mL), dried over anhydrous Na_2_SO_4_, filtered, and concentrated at reduced pressure. The residue was purified via silica gel column chromatography (petroleum ether/EtOAc = 20:1) to afford aldehyde **3** as a yellow oil (0.29 g, 61%). The aldehyde **3** was unstable so it did not characterize and was used in the next step immediately.

### 3.6. (3S,5R,E)-Benzyl 3-hydroxy-5-((4-methoxybenzyl)oxy)-8-methylundeca-8,10-dienoate *(**1**)*

To a solution of Ti(O^i^Pr)_4_ (10.1 mL, 33.8 mmol) in toluene (30 mL), TiCl_4_ (3.37 mL, 30.7 mmol) was added dropwise. The solution was stirred at ambient temperature for 30 min, and the resultant 1 M Ti(O^i^Pr)_2_Cl_2_ solution was used in the next step.

The above freshly prepared Ti(O^i^Pr)_2_Cl_2_ (2.08 mL, 2.08 mmol, 0.6 equiv) solution was cooled to −78 °C to yield a milky white slurry. This slurry was then treated with a solution of aldehyde **3** (1.0 g, 3.47 mmol, 1 equiv) in toluene (25 mL) via a cannula over the course of 10 min at −78 °C. The resultant pale yellow homogeneous solution was stirred at −78 °C for 15 min before being treated with a solution of the ((1-(benzyloxy)vinyl)oxy)trimethylsilane **2b** (4.63 g, 20.8 mmol, 6.0 equiv, prepared following [14]) in toluene (6 mL) via a cannula. The bright yellow reaction mixture was then stirred at −78 °C for another 40 min before being quenched with a saturated aqueous NaHCO_3_ solution (20 mL) when it was observed via TLC that compound **3** was consumed. The mixture was warmed to room temperature and extracted with DCM (3 × 50 mL). The organic extracts were combined, washed with brine, dried over anhydrous Na_2_SO_4_, filtered, and concentrated in vacuo. The residue was purified via flash chromatography (petroleum ether/EtOAc = 10:1) to yield compound **1** (1.09 g, 72%) as a yellow oil.

[α${]}_{D}^{25}$ = +4.1 (*c* = 1, CHCl_3_); ^1^H NMR (400 MHz, CDCl_3_) δ 7.41–7.35 (m, 5H), 7.28 (d, *J* = 7.1 Hz, 2H), 6.88 (d, *J* = 8.3 Hz, 2H), 6.59 (dt, *J* = 16.9 Hz, 10.6 Hz, 1H), 5.86 (d, *J* = 10.8 Hz, 1H), 5.17 (s, 2H), 5.12 (d, *J* = 16.8, 1H), 5.02 (d, *J* = 10.2 Hz, 1H), 4.54–4.45 (m, 2H), 4.41–4.31 (m, 1H), 3.81 (s, 3H), 3.75–3.70 (m, 1H), 3.39 (brs, 1H), 2.59–2.50 (m, 2H), 2.13–2.09 (m, 2H), 1.78 (s, 3H), 1.83–1.60 (m, 4H); ^13^C NMR (CDCl_3_, 100 MHz) δ 172.2, 159.2, 138.9, 135.6, 133.2, 130.3, 129.5, 128.5, 128.2, 128.2, 125.6, 114.9, 113.8, 75.6, 71.0, 66.3, 65.2, 55.2, 41.8, 39.9, 35.2, 31.8, 16.6. HRMS (ESI): *m*/*z* calcd. for C_27_H_34_NO_5_Na [M + Na]^+^ 461.2298, found 461.2302.

### 3.7. Ieodomycin B

To a solution of compound **1** (0.25 g, 0.57 mmol, 1 equiv) in toluene (15 mL), TFA (0.3 mL, 5.1 mmol, 7.0 equiv) was added dropwise at 0 °C and stirred at 0 °C for 30 min. When it was observed via TLC that compound **1** was consumed, the reaction was quenched with a saturated aqueous NaHCO_3_ solution (20 mL). The mixture was warmed to room temperature and extracted with DCM (3 × 20 mL). The organic extracts were combined, washed with brine, dried over anhydrous Na_2_SO_4_, filtered, and concentrated in vacuo. The residue was purified via flash chromatography (petroleum ether/EtOAc = 1:1) to afford the desired natural product, ieodomycin B (60 mg, 50%), as a light yellow oil.

[α${]}_{D}^{25}$ = +22.6 (*c* = 1, CHCl_3_ ); ^1^H NMR (400 MHz, CDCl_3_): δ 6.53 (ddd, *J* = 16.8, 10.4, 10.4 Hz, 1H), 5.84 (d, *J* = 10.4 Hz, 1H), 5.09 (d, *J* = 16.8 Hz, 1H), 4.98 (d, *J* = 10.0 Hz, 1H), 4.28–4.02 (m, 2H), 2.85 (dd, *J* = 16.8 Hz, 3.6 Hz, 1H), 2.44 (dd, *J* = 16.8 Hz, 7.2 Hz, 1H), 2.32–2.12 (m, 3H), 1.88–1.79 (m, 2H), 1.73 (s, 3H), 1.61–1.51 (m, 1H). ^13^C NMR (CDCl_3_, 100 MHz) δ 171.4, 137.5, 132.9, 126.2, 115.4, 76.7, 63.4, 39.3, 37.5, 34.7, 33.4, 16.5. HRMS (ESI): *m*/*z* calcd. for C_12_H_18_O_3_Na [M + Na]^+^ 233.1148, found 233.1149.

## 4. Conclusions

In conclusion, we have obtained ieodomycin B from commercially available geraniol via a concise route. A diastereoselective chelation-controlled Mukaiyama aldol reaction was used to construct the *trans*-diol, and a TFA-promoted one-pot reaction was used to accomplish the deprotection and lactonization steps. This route is an alternative approach to ieodomycin B synthesis. The high *anti*-selectivity of the Mukaiyama aldol addition, which results in the formation of the *anti*-diol product, is assumed to arise from the β-chelation of titanium cation by the oxygens of the aldehyde and the PMB-protected ether. Further application of such approach in the preparation of analogues of ieodomycins is now in progress.

## Figures and Tables

**Table 1:** Deprotection and lactonization conditions for the preparation of ieodomycin B.

Entry	Reagent	Solvent	Temperature	Yield
1	*p*-TsOH	MeOH	70 °C	- ^1^
2	PPTS	MeOH	reflux	- ^1^
3	DDQ	DCM	−78 °C	- ^1^
4	CAN	DCM	−78 °C	- ^1^
5	TFA	DCM	0 °C	50%

^1^ Without isolation, the yield for the fuzzy spots in thin-layer chromatography (TLC) was not calculated.

## Supplementary Materials

The following are available online at www.mdpi.com/1660-3397/15/1/17/s1: Figure S1: ^1^H NMR spectra of Compound **4**, Figure S2: ^13^C NMR spectra of Compound **4**, Figure S3: ^1^H NMR spectra of Compound **11**, Figure S4: ^13^C NMR spectra of Compound **11**, Figure S5: ^1^H NMR spectra of Compound **12**, Figure S6: ^13^C NMR spectra of Compound **12**, Figure S7: ^1^H NMR spectra of Compound **1**, Figure S8: ^13^C NMR spectra of Compound **1**, Figure S9: ^1^H NMR spectra of ieodomycin B, Figure S10: ^13^C NMR spectra of Ieodomycin B, Figure S11: MS spectra of Compound **11**: HRMS (ESI): *m*/*z* calcd. for C_12_H_21_NO_3_Na [M + Na]^+^ 250.1414, found 250.1416, Figure S12: MS spectra of Compound **12**: HRMS (ESI): *m*/*z* calcd. for C_20_H_29_NO_4_Na [M + Na]^+^ 370.1989, found 370.1989, Figure S13: MS spectra of Compound **1**: HRMS (ESI): *m*/*z* calcd. for C_27_H_34_NO_5_Na [M + Na]^+^ 461.2298, found 461.2302, Figure S14: MS spectra of ieodomycin B: HRMS (ESI): *m*/*z* calcd. for C_12_H_18_O_3_Na [M + Na]^+^ 233.1148, found 233.1149.

## References

[1] Blunt J.W., Copp B.R., Keyzers R.A., Munro M.H.G., Prinsep M.R. (2013). Marine natural products. Nat. Prod. Rep..

[2] Demain A.L., Vaishnav P. (2011). Natural products for cancer chemotherapy. Microb. Biotechnol..

[3] Sawadogo W.R., Schumacher M., Teiten M.H., Cerella C., Dicato M., Diederich M. (2013). A survey of marine natural compounds and their derivatives with anti-cancer activity reported in 2011. Molecules.

[4] Garcia A., Bocanegra-Garcia V., Palma-Nicolas J.P., Rivera G. (2012). Recent advances in antitubercular natural products. Eur. J. Med. Chem..

[5] Mojid Mondol M.A., Kim J.H., Lee M.A., Tareq F.S., Lee H.-S., Lee Y.-J., Shin H.J. (2011). Ieodomycins A–D, Antimicrobial Fatty Acids from a Marine *Bacillus* sp.. J. Nat. Prod..

[6] Salunkhe V.T., Bhosale S., Punde P., Bhuniya D., Koul S. (2013). Stereo-controlled total syntheses of ieodomycins A and B using d-glucose based chiral pool approach. Tetrahedron Lett..

[7] Reddy E.N., Krishnaiah A., Rao T.P. (2013). Stereoselective total synthesis of Ieodomycin A and B. Tetrahedron Asymmetry.

[8] Rao N.N., Meshram H.M. (2013). Protection-free, short, and stereoselective synthesis of ieodomycin A and B. Tetrahedron Lett..

[9] Das S., Goswami R.K. (2013). Stereoselective Total Synthesis of Ieodomycins A and B and Revision of the NMR Spectroscopic Data of Ieodomycin B. J. Org. Chem..

[10] Wang X.J., Zhang J.T., Wang L.P., Chen S.P., Tang L.J., Huang S.P. (2015). Concise Total Synthesis of Ieodomycin A and B. Synth. Commun..

[11] Evans D.A., Dart M.J., Duffy J.L., Yang M.G. (1996). A Stereochemical Model for Merged 1,2- and 1,3-Asymmetric Induction in Diastereoselective Mukaiyama Aldol Addition Reactions and Related Processes. J. Am. Chem. Soc..

[12] Perry N.B., Blunt W.B., McCombs J.D., Munro M.H.G. (1986). Dramatic Effects of Oxygen Substituents on 1,3-Asymmetric Induction in Additions of Allyltriphenylstannane to β-Alkoxy Aldehydes: A Chemical and Spectroscopic Investigation. J. Org. Chem..

[13] Keck G.E., Castellino S. (1986). On the Origins of Stereoselectivity in “Chelation Controlled” Nucleophilic Additions to β-Alkoxy Aldehydes: Solution Structures of Lewis Acid Complexes via NMR Spectroscopy. J. Am. Chem. Soc..

[14] Kiyooka S.-I., Matsumoto S., Shibata T., Shinozaki K.-I. (2010). Platinum (II) complex-catalyzed enantioselective aldol reaction with ketene silyl acetals in DMF at room temperature. Tetrahedron.

