# Supplementary material for: Asymmetric Total Synthesis of Ieodomycin B
Source: Mar Drugs. 2017 Jan 18;15(1):17. doi: 10.3390/md15010017 (PMC5295237; doi:10.3390/md15010017)
Supplement: Supplementary file 1 [file marinedrugs-15-00017-s001.docx]

Supplementary Materials: Asymmetric Total Synthesis of Ieodomycin B

Shuangjie Lin, Jianting Zhang, Zhibin Zhang, Tianxiang Xu, Shuangping Huang and
Xiaoji Wang


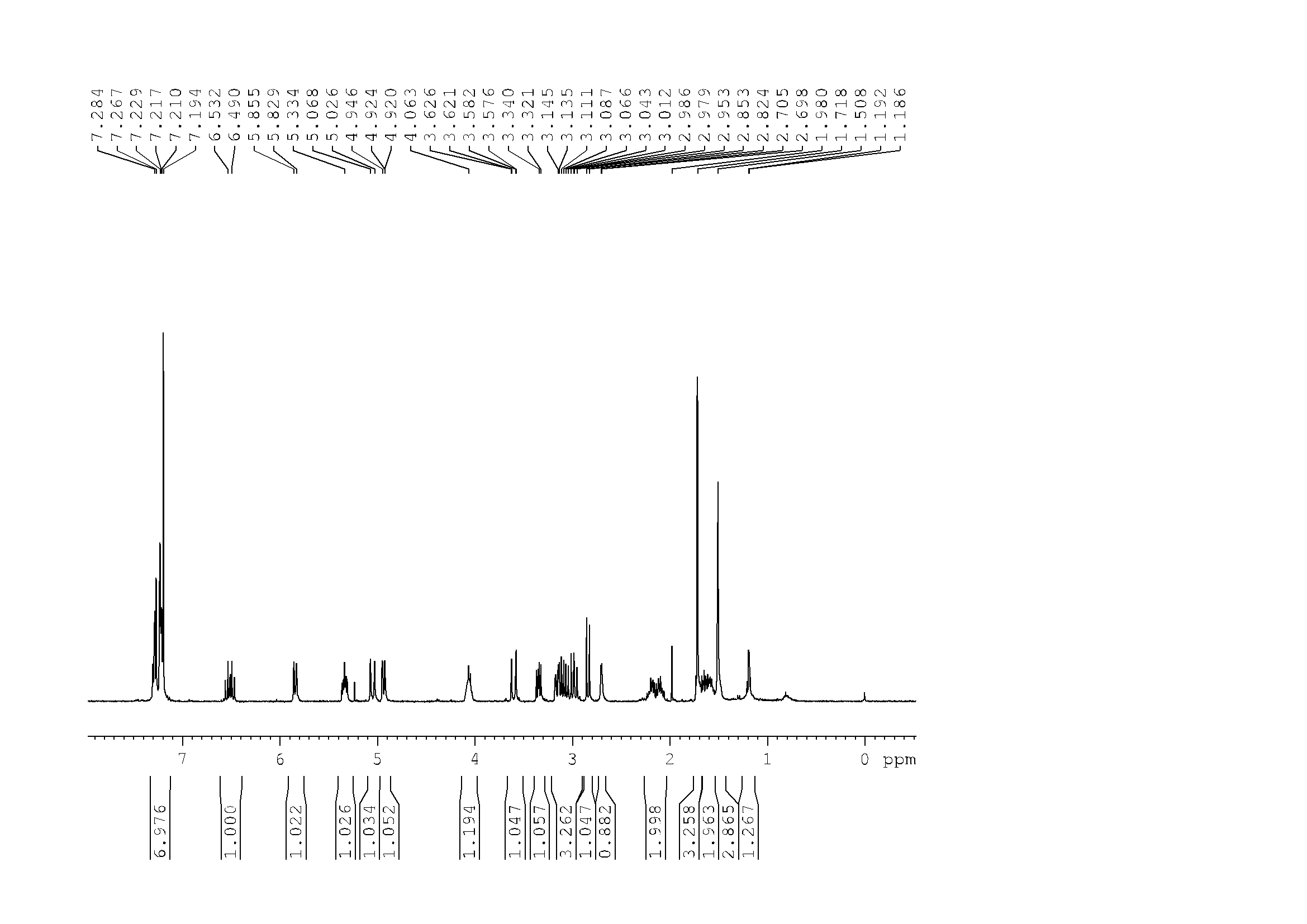


**Figure S1.** ^1^H NMR spectra of Compound **4**.


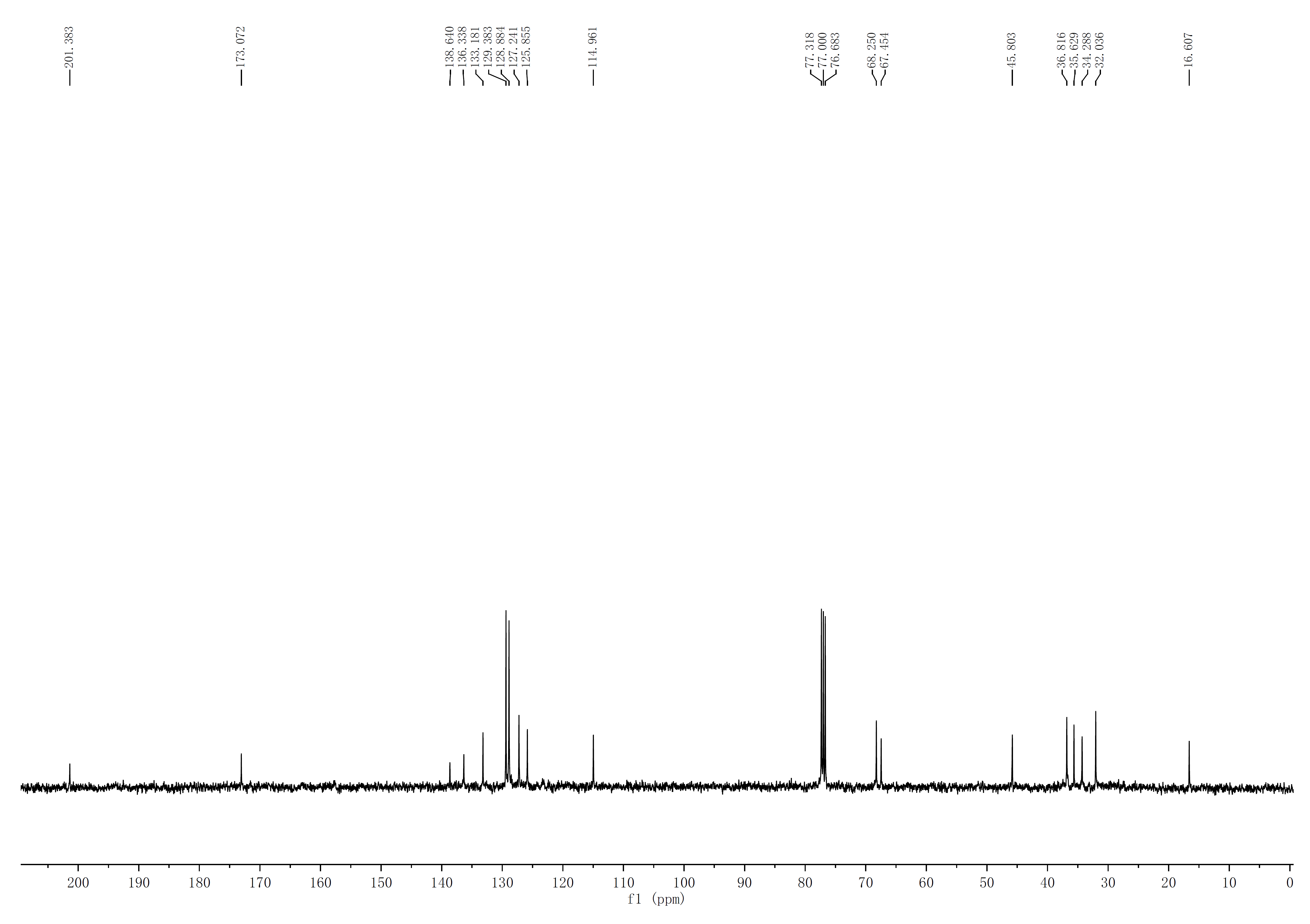


**Figure S2.** ^13^C NMR spectra of Compound **4**.

**
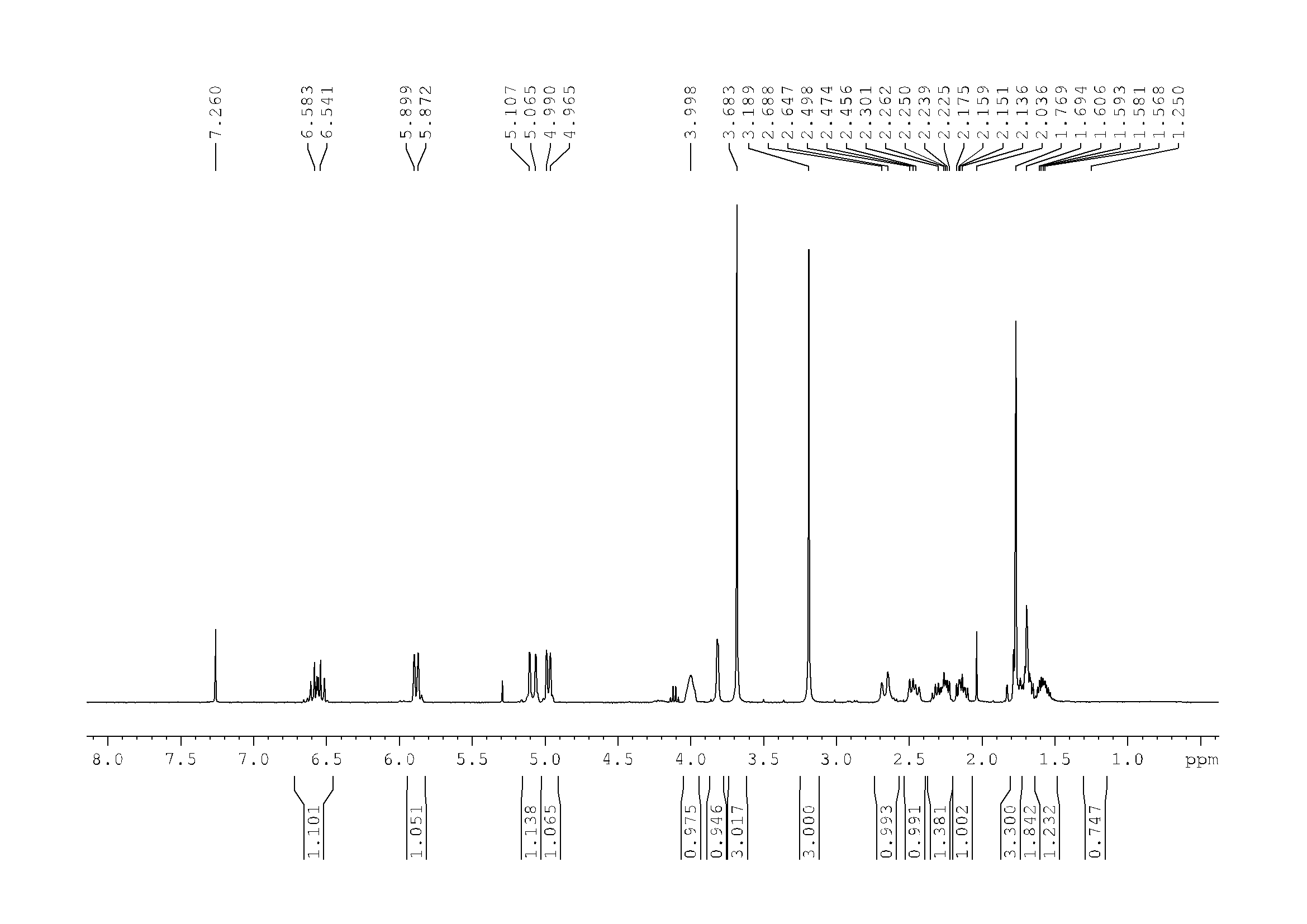
**

**Figure S3.** ^1^H NMR spectra of Compound **11**.


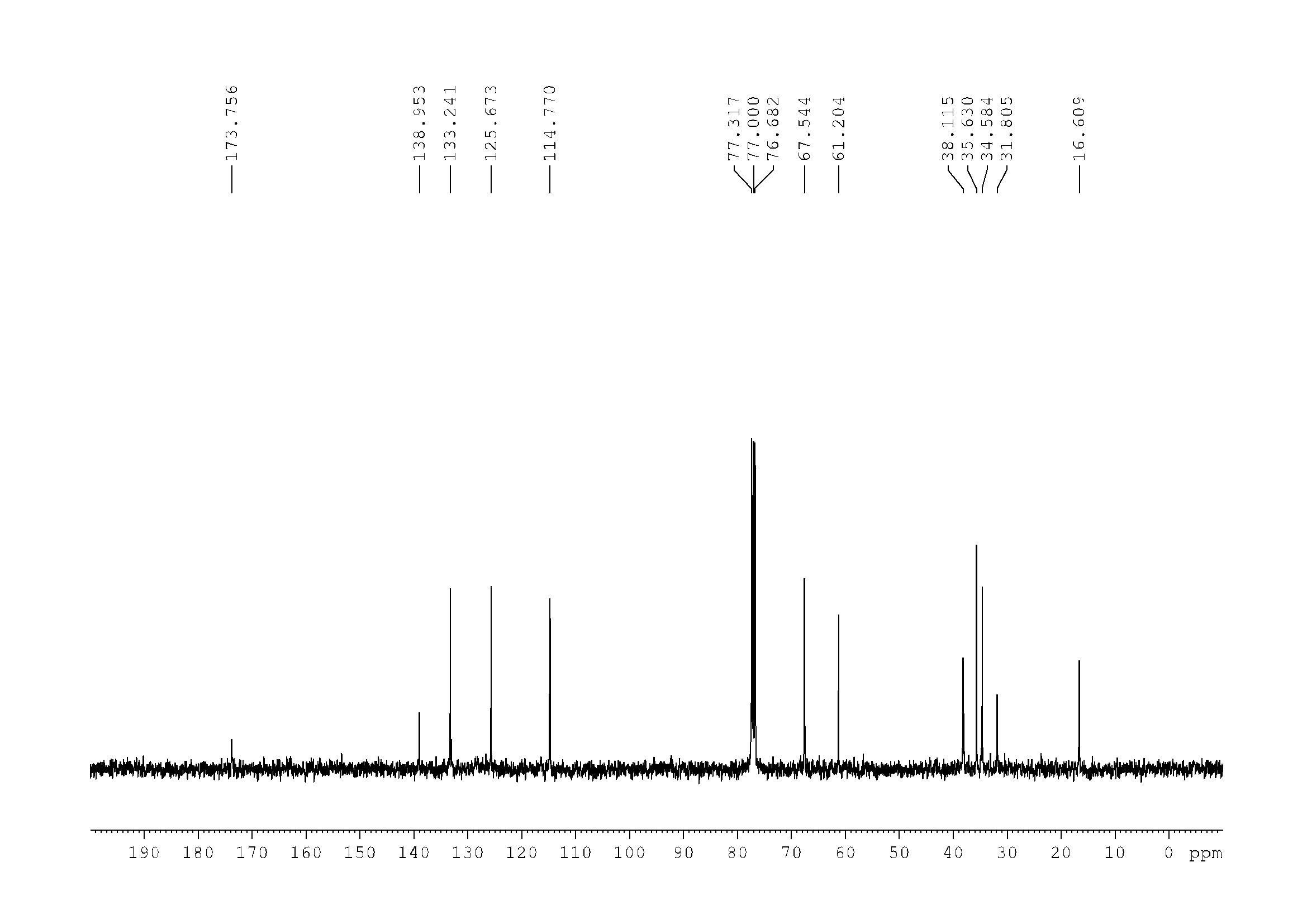


**Figure S4.** ^13^C NMR spectra of Compound **11**.


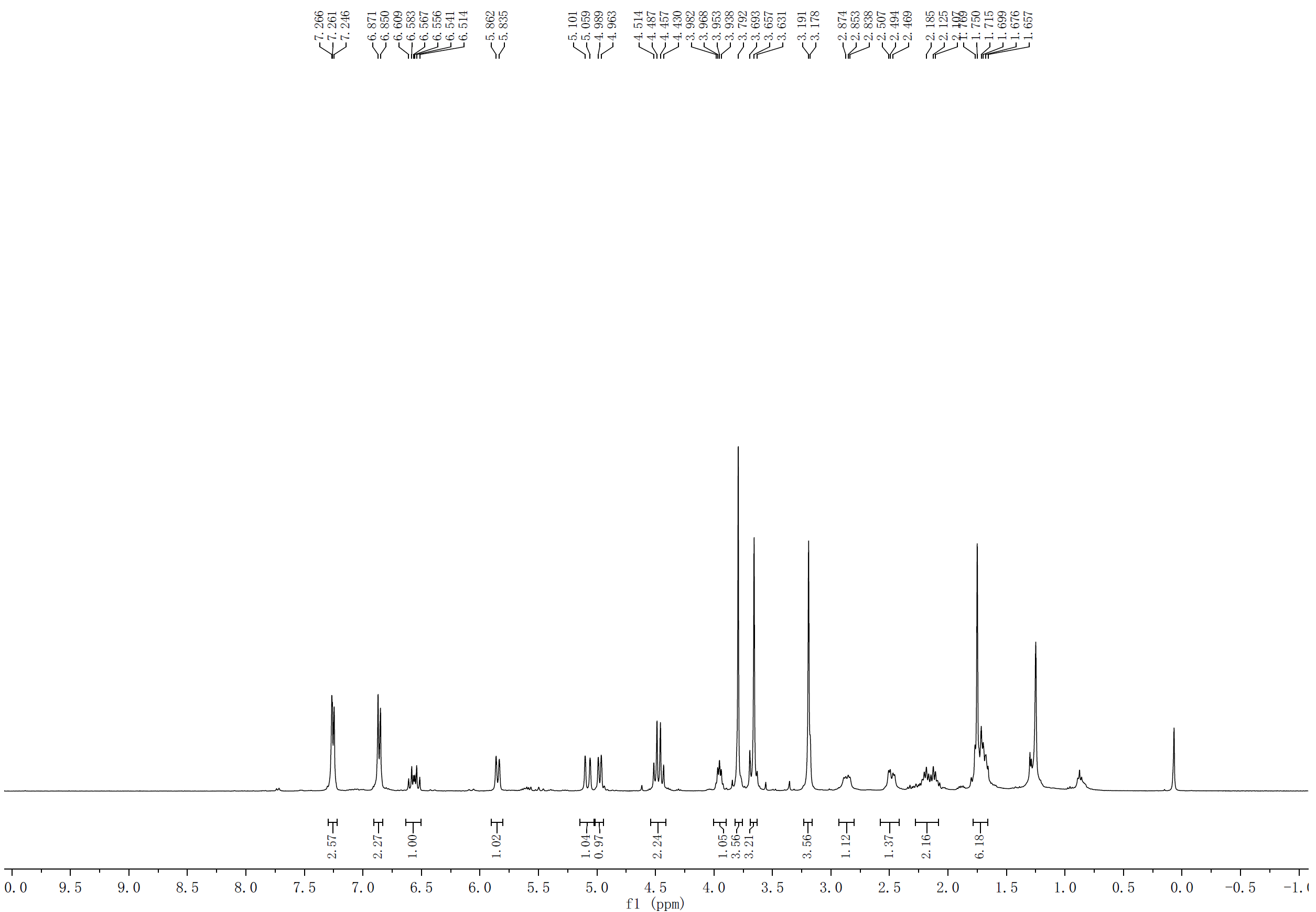


**Figure S5.** ^1^H NMR spectra of Compound **12**.


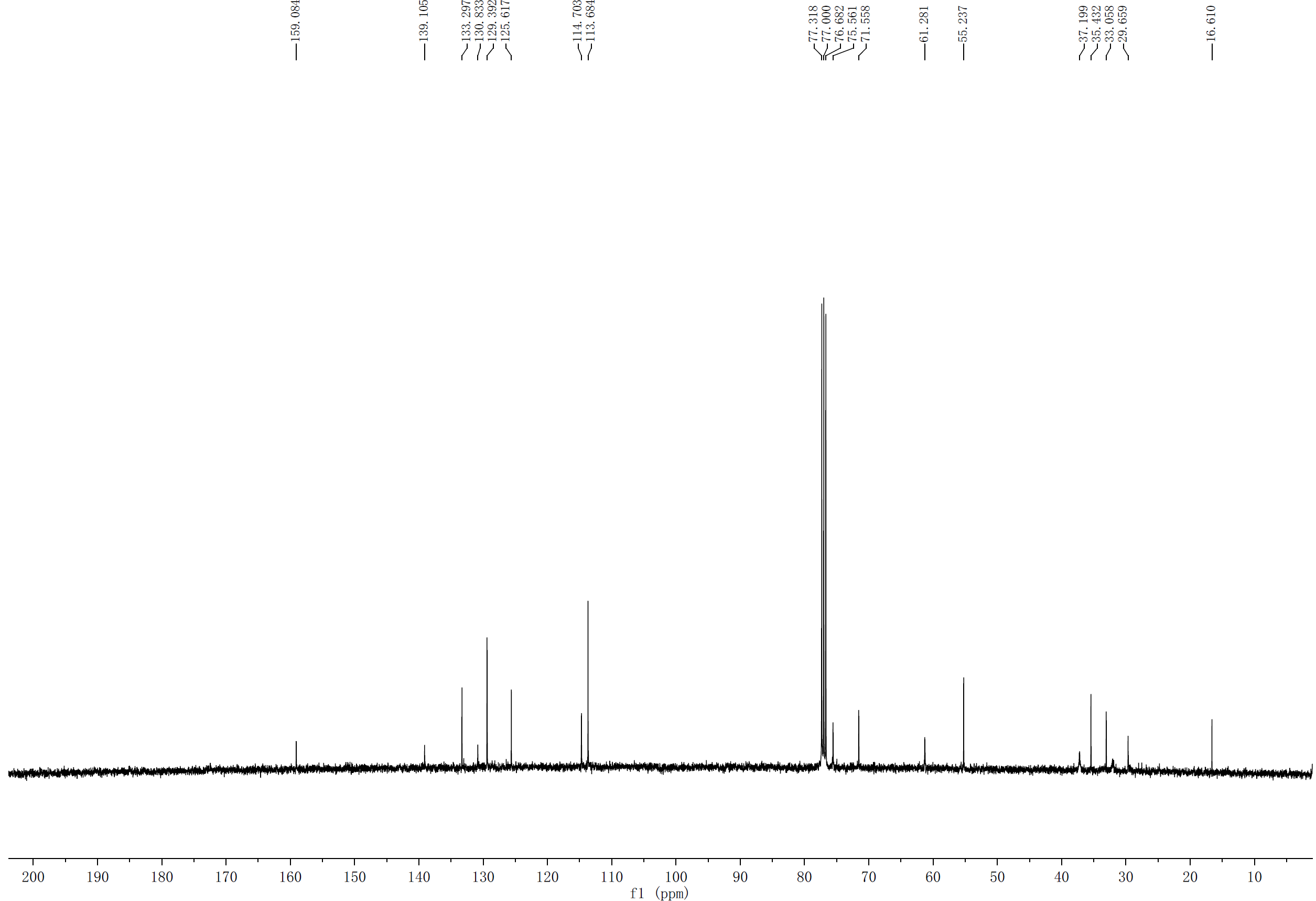


**Figure S6.** ^13^C NMR spectra of Compound **12**.


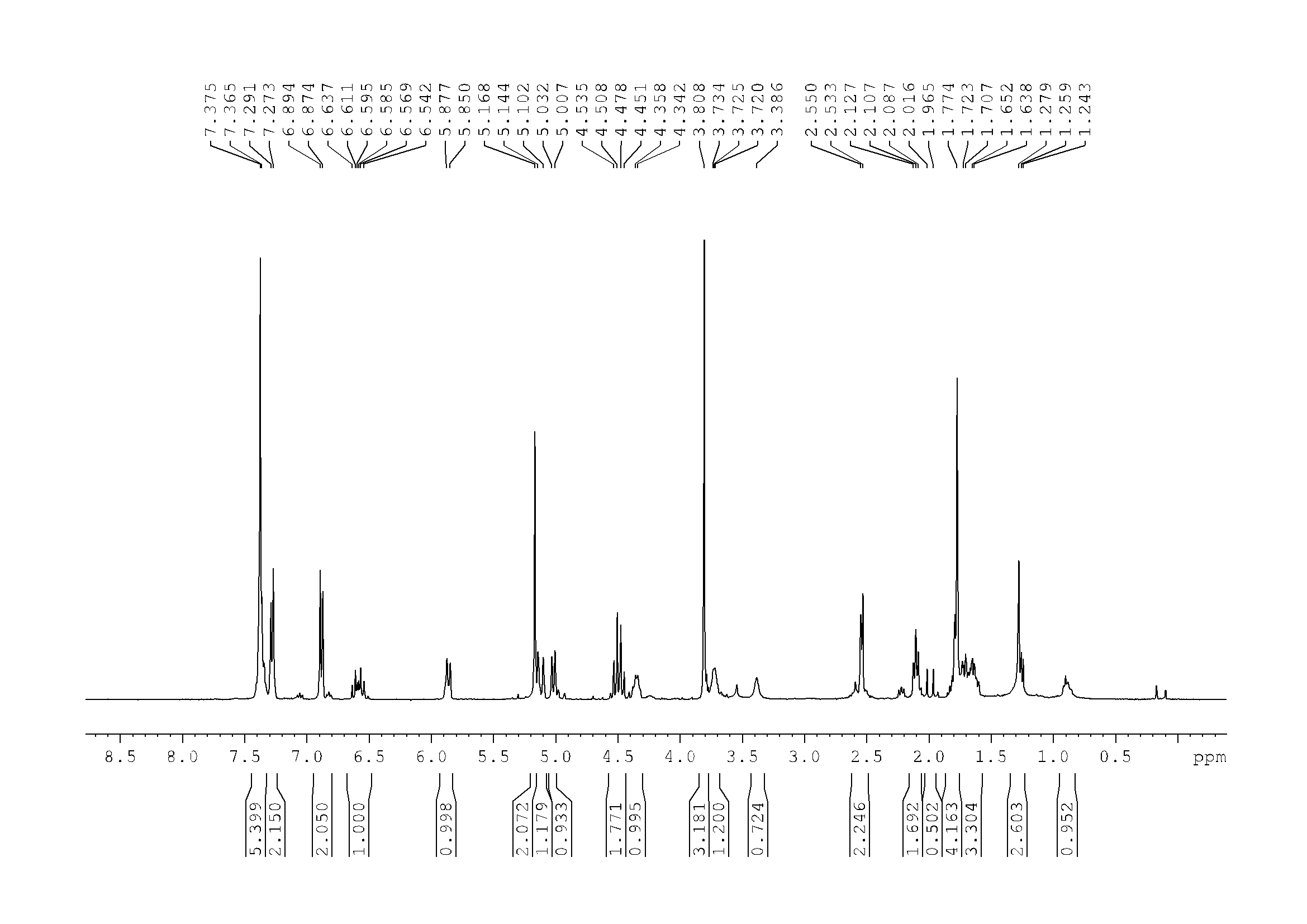


**Figure S7.** ^1^H NMR spectra of Compound **1**.


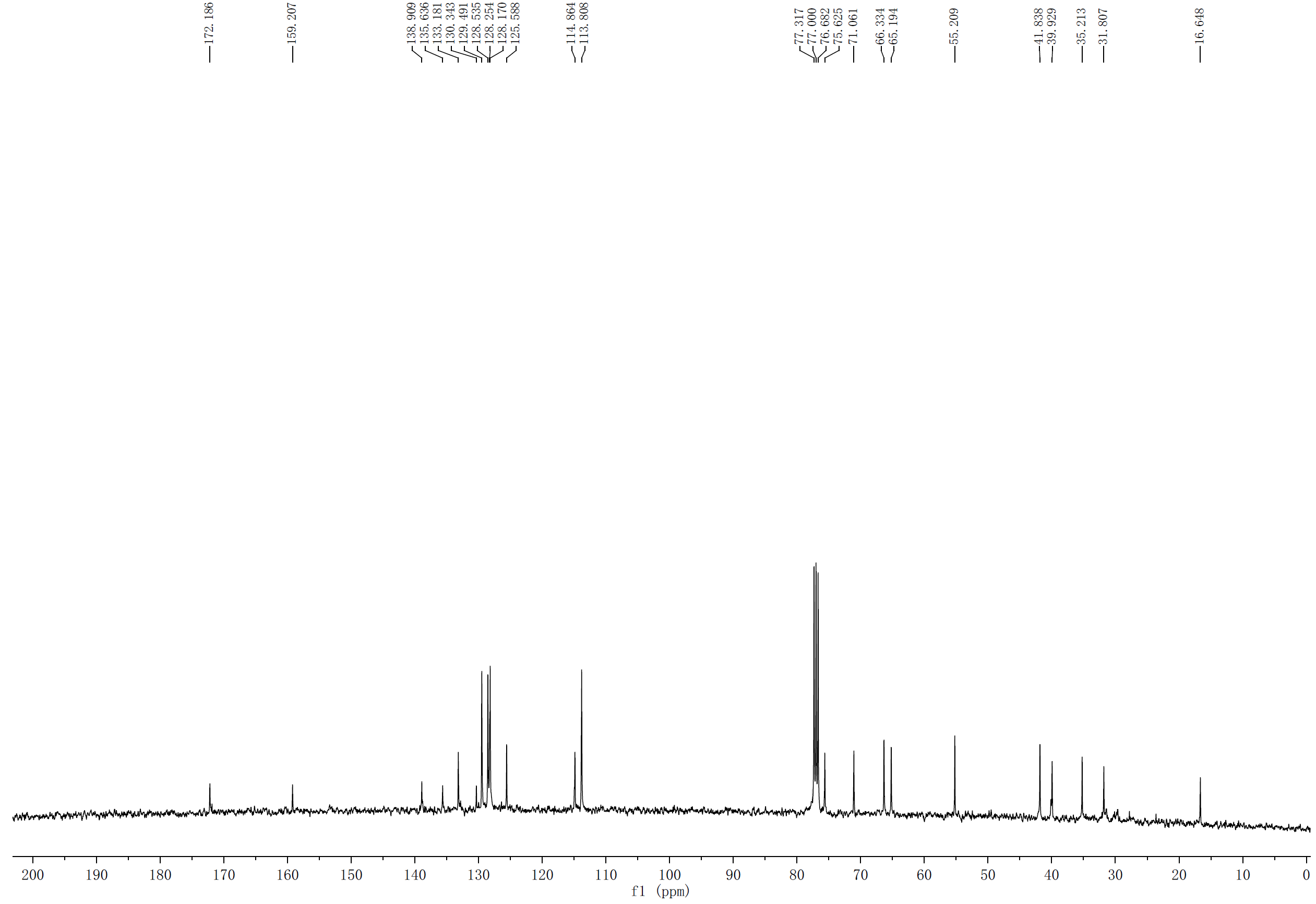


**Figure S8.** ^13^C NMR spectra of Compound **1**.


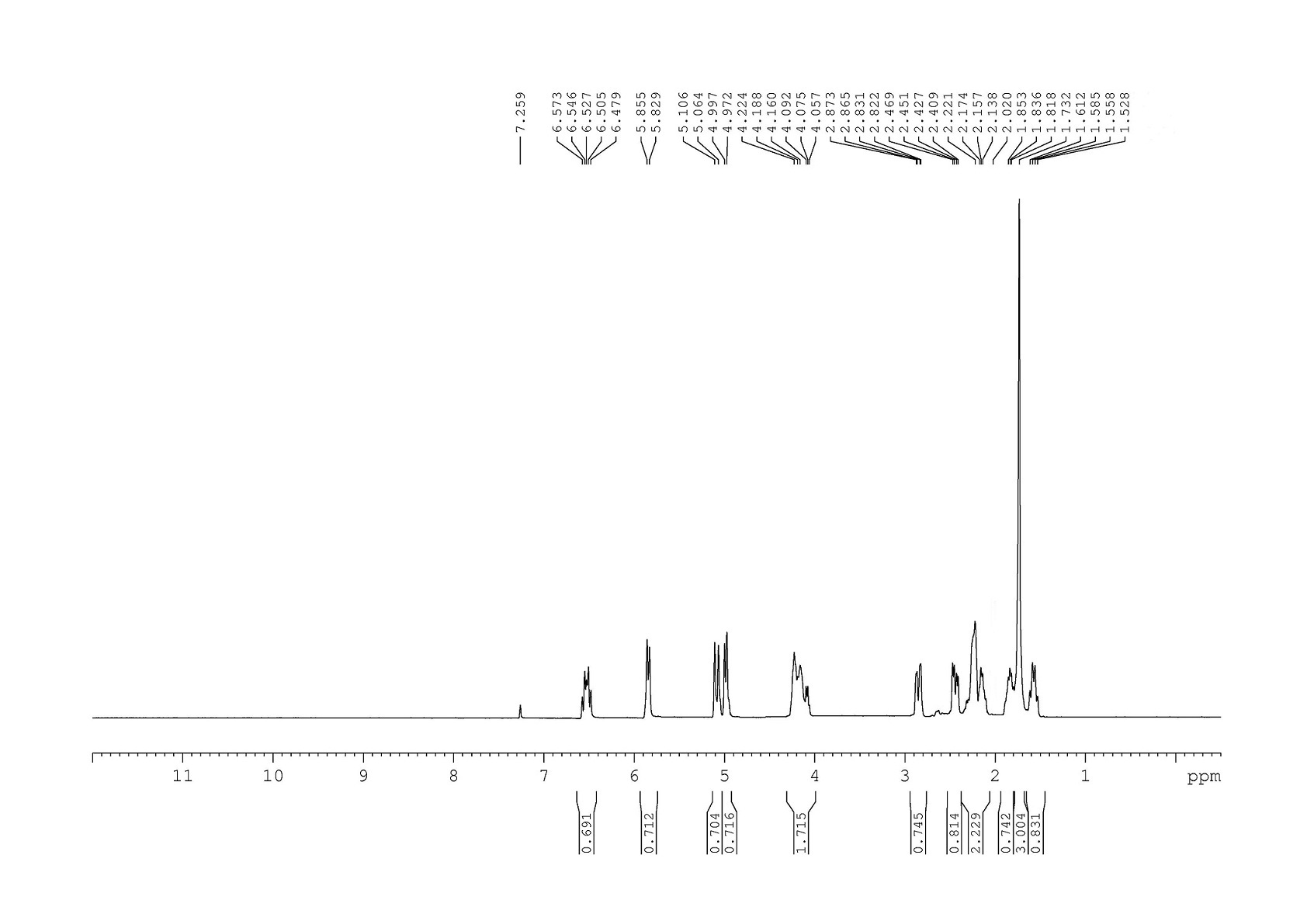


**Figure S9.** ^1^H NMR spectra of ieodomycin B.


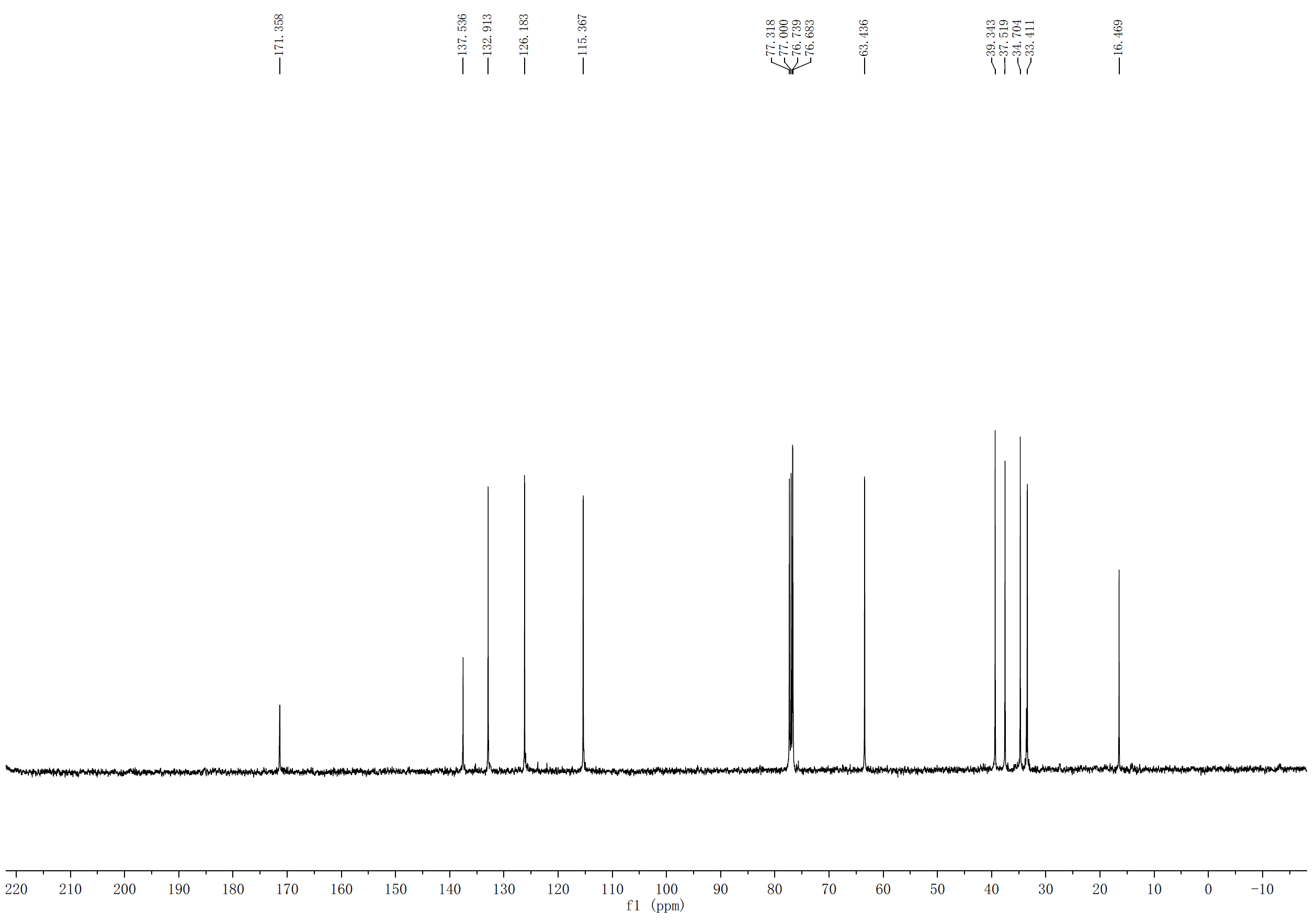


**Figure S10.** ^13^C NMR spectra of ieodomycin B.

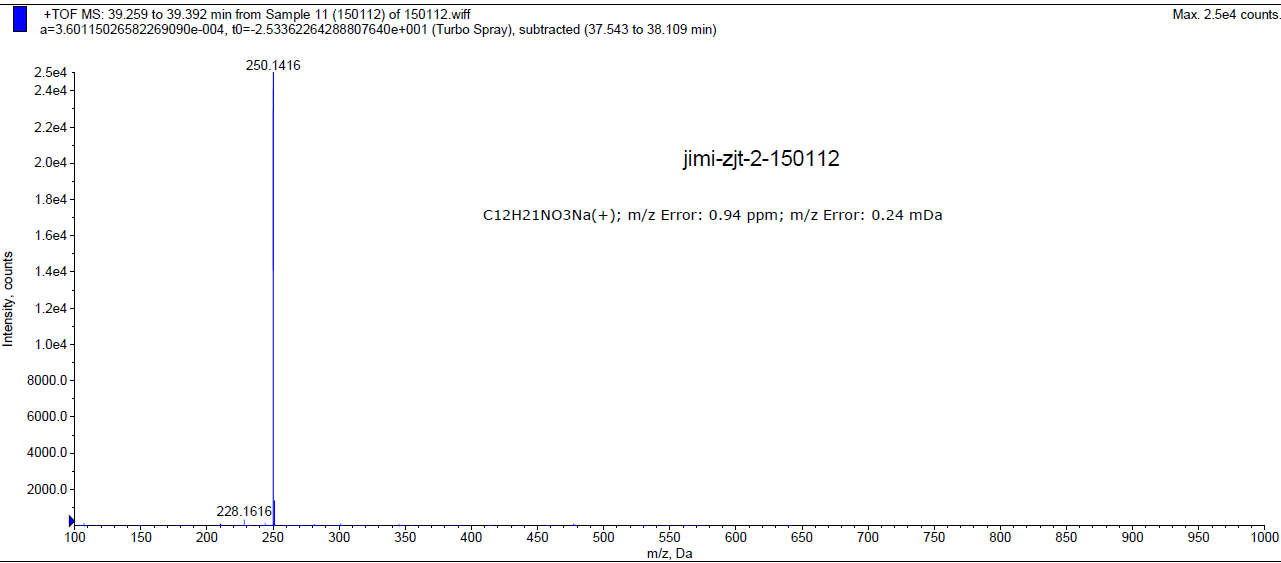


**Figure S11.** MS spectra of Compound **11**: HRMS (ESI): *m*/*z* calcd. for C_12_H_21_NO_3_Na [M + Na]^+^  250.1414, found 250.1416.

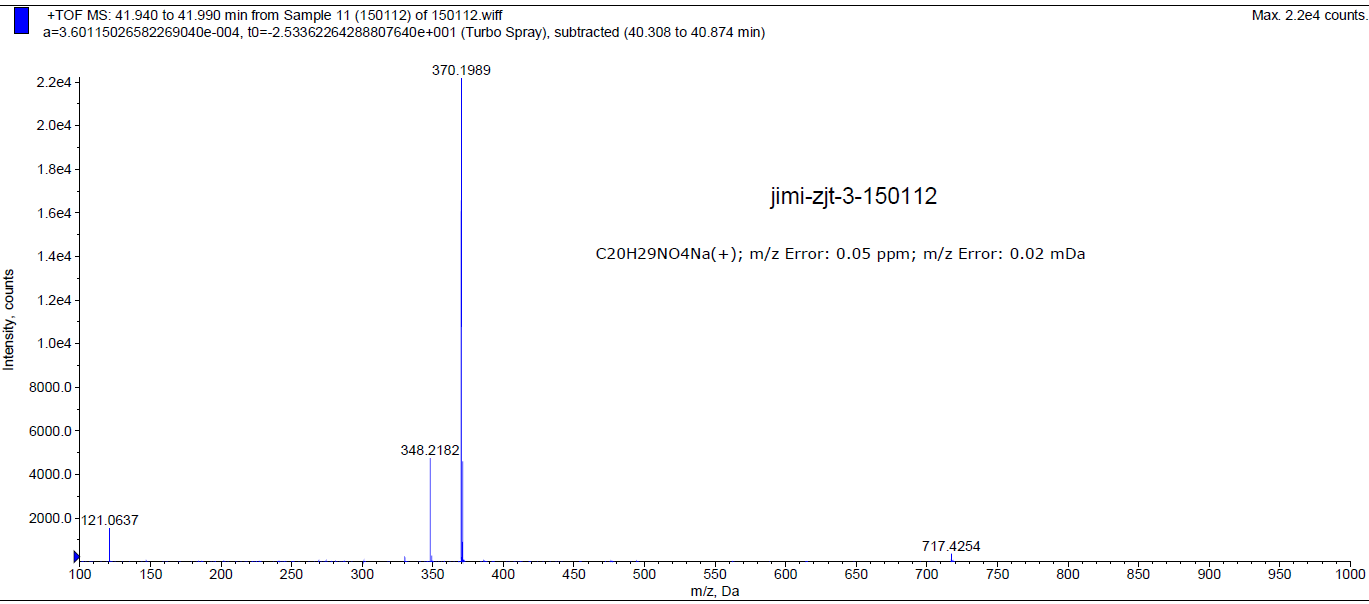


**Figure S12.** MS spectra of Compound **12**: HRMS (ESI): *m*/*z* calcd. for C20H29NO4Na [M + Na]^+^ 370.1989, found 370.1989.

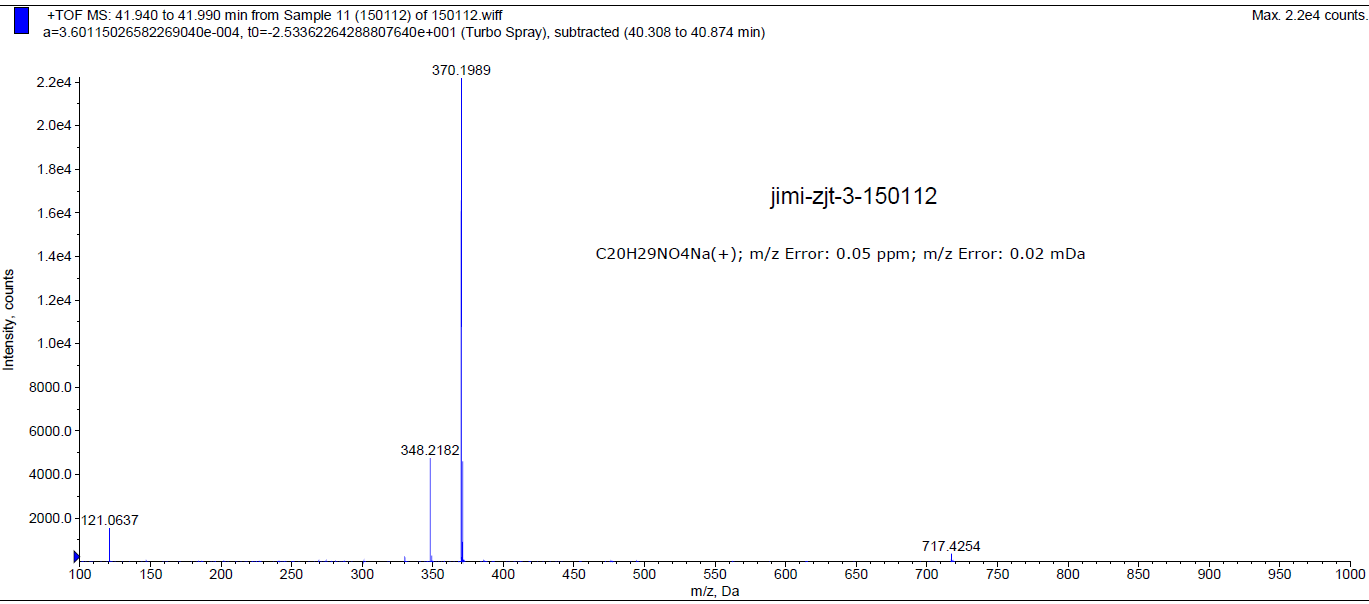


**Figure S13.** MS spectra of Compound **1**: HRMS (ESI): *m*/*z* calcd. for C_27_H_34_NO_5_Na [M + Na]^+^ 461.2298, found 461.2302.


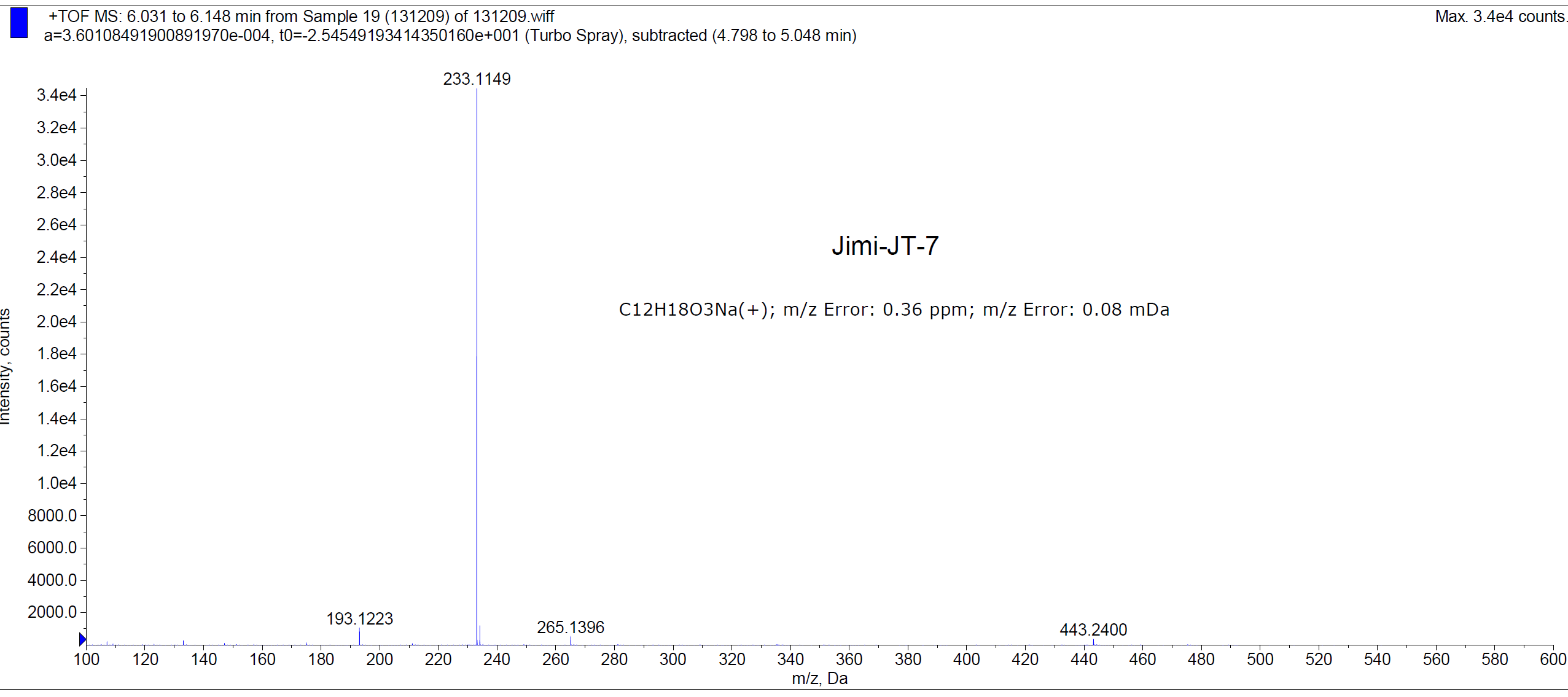


**Figure S14.** MS spectra of ieodomycin B: HRMS (ESI): *m*/*z* calcd. for C_12_H_18_O_3_Na [M + Na]^+^ 233.1148, found 233.1149.
